# 

**Published:** 1899-12

**Authors:** 


### CONTENTS.

COMMUNICATIONS.

Sulphate of Magnesia—Abstract ........ 541

PROCEEDINGS.

National Dental Association, Niagara Falls, August 1–4, 1899........ 544
Cements........ 544
Some Phases of the Cement Question ........ 545
Susceptibility and Immunity to Dental Caries........ 547
Management of Children's Teeth........ 548

SELECTIONS.

No Quack Advertisements........ 556
Evolution of Decay........ 557
Pure-Food Legislation ........ 567
Dangers of Colds ........ 569
A Banquet Without Wine........ 570
Antidotal Properties of Permanganate of Potassium ........ 571
Reception to the Medical Students ........ 571
A Local and a National Reproach ........ 572
The Russian Government has Forbidden the Sale of a Supposed Herbal Remedy........ 572
The New Anatomy ........ 573
New Application of Iodin ........ 573
The Immorality of "Christian Science." ........ 574
Medical Women ........ 574
Morphinism Among Physicians........ 575
Slight Rises of Temperature after an Operation........ 575
Report of Law Committee ........ 575

NOTICES.

Toledo Dental Society ........ 579

EDITORIAL.

The National School of Dental Technics ........ 579

